# Common Variants in the Glycerol Kinase Gene Reduce Tuberculosis Drug Efficacy

**DOI:** 10.1128/mBio.00663-19

**Published:** 2019-07-30

**Authors:** Michelle M. Bellerose, Seung-Hun Baek, Chuan-Chin Huang, Caitlin E. Moss, Eun-Ik Koh, Megan K. Proulx, Clare M. Smith, Richard E. Baker, Jong Seok Lee, Seokyong Eum, Sung Jae Shin, Sang-Nae Cho, Megan Murray, Christopher M. Sassetti

**Affiliations:** a Department of Microbiology and Physiological Systems, University of Massachusetts Medical School, Worcester, Massachusetts, USA; b Department of Microbiology, Institute for Immunology and Immunological Diseases, Yonsei University College of Medicine, Seoul, South Korea; c Department of Global Health and Social Medicine, Harvard Medical School, Boston, Massachusetts, USA; d International Tuberculosis Research Center, Changwon, South Korea; New York University School of Medicine; Harvard University; Cornell University

**Keywords:** *Mycobacterium tuberculosis*, antibiotic resistance, genetics

## Abstract

Despite the administration of multiple drugs that are highly effective *in vitro*, tuberculosis (TB) treatment requires prolonged drug administration and is confounded by the emergence of drug-resistant strains. To understand the mechanisms that limit antibiotic efficacy, we performed a comprehensive genetic study to identify Mycobacterium tuberculosis genes that alter the rate of bacterial clearance in drug-treated mice. Several functionally distinct bacterial genes were found to alter bacterial clearance, and prominent among these was the *glpK* gene that encodes the glycerol-3-kinase enzyme that is necessary for glycerol catabolism. Growth on glycerol generally increased the sensitivity of M. tuberculosis to antibiotics *in vitro*, and *glpK*-deficient bacteria persisted during antibiotic treatment *in vivo*, particularly during exposure to pyrazinamide-containing regimens. Frameshift mutations in a hypervariable homopolymeric region of the *glpK* gene were found to be a specific marker of multidrug resistance in clinical M. tuberculosis isolates, and these loss-of-function alleles were also enriched in extensively drug-resistant clones. These data indicate that frequently observed variation in the *glpK* coding sequence produces a drug-tolerant phenotype that can reduce antibiotic efficacy and may contribute to the evolution of resistance.

## Abstract (precis)

TB control is limited in part by the length of antibiotic treatment needed to prevent recurrent disease. To probe mechanisms underlying survival under antibiotic pressure, we performed a genetic screen for M. tuberculosis mutants with altered susceptibility to treatment using the mouse model of TB. We identified multiple genes involved in a range of functions which alter sensitivity to antibiotics. In particular, we found glycerol catabolism mutants were less susceptible to treatment and that common variation in a homopolymeric region in the *glpK* gene was associated with drug resistance in clinical isolates. These studies indicate that reversible high-frequency variation in carbon metabolic pathways can produce phenotypically drug-tolerant clones and have a role in the development of resistance.

## INTRODUCTION

The currently used multidrug chemotherapy regimen for tuberculosis (TB) was developed in a series of clinical trials in the 1980s (1) and remains the standard of care for this disease (2). Infections with drug-sensitive strains of Mycobacterium tuberculosis are treated with a 6-month regimen that includes four drugs, isoniazid (INH), rifampin (RIF), pyrazinamide (PZA), and ethambutol (EMB). While this regimen cures 90% of drug-sensitive cases, the long period over which antibiotics must be administered represents a major limitation. Not only is a complete regimen difficult to deliver, but even in clinical trial settings, incomplete sterilization leads to relapse in a significant fraction of patients (3). The emergence of drug-resistant strains of M. tuberculosis further confounds therapy and necessitates even longer regimens with less effective drugs.

The factors that necessitate this extended drug regimen for TB remain difficult to dissect because the *in vitro* efficacy of individual drugs does not predict their effect during infection. For example, PZA is critical for sterilizing an infected host, but it has very modest activity *in vitro*, where it may act via different mechanisms (4). Conversely, both INH and RIF cause relatively rapid cell death *in vitro* but kill bacteria much more slowly during infection. As a result, virtually all TB drug regimens kill bacteria at a lower rate during infection than they do in axenic culture. Two general mechanisms have been proposed to explain the generally drug-tolerant phenotype that is observed during infection. Growth in mammalian tissue triggers changes in mycobacterial gene expression and metabolism that can reduce drug efficacy (5–7). In addition, a number of distinct stochastically generated subpopulations have been observed, which arise either via asymmetric cell division (8) or nonheritable regulatory events (9). Some of these subpopulations are relatively insensitive to antibiotics *in vitro* and could prolong the treatment period necessary for sterilization. While none of these mechanisms involve heritable genetic changes, many other bacteria rely on high-frequency reversible genetic variation to produce subpopulations that are tolerant to environmental insults (10). This process of phase variation generally relies on specific DNA sequences, such as homopolymeric regions, that are subject to frequent mutation. While phase variation has been observed in several mycobacterial species (11, 12), it has not been specifically characterized in M. tuberculosis, and its potential role in determining drug efficacy is unknown.

To understand the processes that determine drug efficacy during infection, we employed two complementary approaches. A forward genetic study identified bacterial functions that alter drug efficacy in mice. In parallel, whole-genome sequence analysis of M. tuberculosis clinical isolates identified genetic variants in candidate genes that are associated with resistance. Together, these approaches defined a variable homopolymeric region in the *glpK* gene that controls glycerol metabolism and drug efficacy. Heritable genetic variation at this site produces a drug-tolerant phenotype that reduces treatment efficacy and is associated with the emergence of resistant clones.

## RESULTS

### Genetic determinants of drug efficacy in the mouse model.

**FIG 1:**
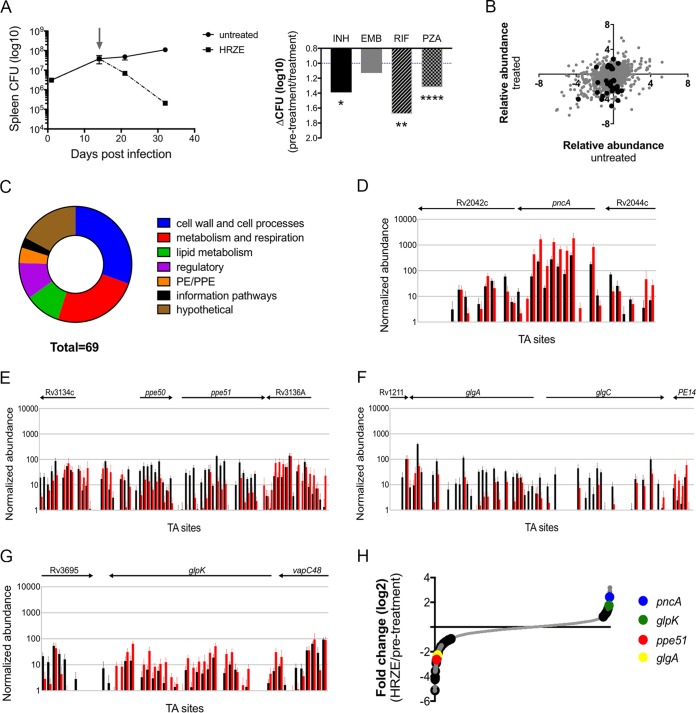
Genetic strategy to define bacterial functions that limit drug efficacy. (A, left) Spleen CFU from BALB/c mice infected with transposon mutant library both untreated (circles) and after HRZE treatment (squares). Antibiotic treatment was started at 14 dpi (indicated by gray arrow). Plotted means from 3 biological replicates with standard deviations are shown. (Right) Change in CFU after treatment with the indicated antibiotic for 5 weeks. The change in CFU between pretreatment and posttreatment samples is presented. Significance was calculated using unpaired *t* test: *, *P* = 0.03; **, *P* = 0.002; ***, *P* = 0.0002; ****, *P* < 0.0001. (B) Relative abundance of individual mutants, measured by log_2_ fold change, in untreated mice (*x* axis) and HRZE-treated mice (*y* axis). Significantly altered mutants after treatment are indicated in black. (C) Functional classes of mutants with altered susceptibility *in vivo*. Classification from Mycobrowser. (D to G) Normalized abundance of mutations in pretreatment (black) and after HRZE treatment (red) at individual TA dinucleotide insertion sites in *pncA* (D), *ppe50-ppe51* (E), *glgA-glgC* (F), and *glpK* (G). Shown are the average numbers of unique sequence reads (*y* axis) plotted versus TA sites (*x* axis). (H) Log_2_ fold change of individual mutants (gray dots) 1 week posttreatment compared to pretreatment. Significantly altered mutants are indicated by black circles.

To specifically define bacterial functions that limit efficacy during infection, we used transposon sequencing (TNseq) to identify mutations that alter bacterial killing. Groups of mice were infected with a nearly saturated library of M. tuberculosis transposon mutants via the intravenous route. After allowing 2 weeks for bacterial growth and the establishment of adaptive immunity, animals were treated with a regimen based on first-line TB chemotherapy, a mixture of INH, RIF, PZA, and EMB (HRZE). Each drug in this regimen was shown to be effective individually at the dose given, and the four-drug mixture reduced organ burden by more than 100-fold after 14 days of treatment (Fig. 1A). To identify mutations with relatively rapid effects on bacterial killing by antibiotics, mutant pools were recovered from the spleen after 1 week of therapy by plating organ homogenates. This analysis was performed in the spleen because this organ contained an adequate bacterial population size to ensure that complexity of the library was maintained throughout the infection. *In vivo*-selected libraries were compared to each other using TNseq, which quantifies the relative abundance of each mutant in a given pool by sequencing all the transposon-chromosome junctions that are present (13).

Mutant pools were collected from three groups of animals. A pretreatment pool was collected immediately before drug administration. One week later, pools from antibiotic-treated or untreated groups were collected. This study design allowed the relative fitness of each bacterial mutant to be assessed in the presence and absence of drug therapy. Pairwise analyses of the treated and untreated pools with the pretreatment library identified distinct sets of genes that altered bacterial representation under each condition (see Table S1 in the supplemental material). As many antibiotics act in a growth rate-dependent manner, we investigated whether bacterial fitness *in vivo* was an important determinant of antibiotic efficacy. When the relative abundance of each mutant in untreated animals was compared with their abundance in time-matched drug-treated mice, we found no global correlation between bacterial fitness in the presence and absence of antibiotic (Fig. 1B). However, several individual mutations were observed that reduced fitness under both conditions. Thus, to more formally focus our study on drug-related phenotypes, we performed a three-way analysis to identify those mutations that alter fitness preferentially in antibiotic-treated animals. This analysis defined 61 mutants that increased, and 8 that reduced, the effect of therapy (Table S2). These mutants corresponded to a variety of functional pathways (Fig. 1C). In several cases mutation resulted in dramatic alterations in fitness after drug exposure, as the relative representation of these mutants under the pre- and posttreatment conditions varied by more than 100-fold.

10.1128/mBio.00663-19.1 TABLE S1 Whole-genome phenotypic profiling of M. tuberculosis mutants. Pairwise analyses of the treated and untreated pools with the pretreatment library. Mean TAs hit, average number of transposon insertion sites sequenced across all comparisons. Mean_hits/TA, average number of unique sequenced templates divided by the TA insertions sites in the gene. Pval, *P* value derived from resampling. Qval, significance adjusting for multiple tests. Gray shading, low-confidence genes (having an average of 3 or fewer transposon insertions across all comparisons). Download Table S1, XLSX file, 0.9 MB. Copyright © 2019 Bellerose et al. 2019 Bellerose et al. This content is distributed under the terms of the Creative Commons Attribution 4.0 International license.

10.1128/mBio.00663-19.2 TABLE S2 Whole-genome phenotypes using 3-way analysis. Three-way analyses of pretreatment, untreated, and treated pools. mean_, average number of unique sequenced templates divided by the TA insertion sites in the gene. Pval, *P* value derived from resampling. Qval, significance adjusting for multiple tests. Download Table S2, XLSX file, 0.5 MB. Copyright © 2019 Bellerose et al. 2019 Bellerose et al. This content is distributed under the terms of the Creative Commons Attribution 4.0 International license.

A number of functions found to alter bacterial fitness in drug-treated mice were already known to impact drug efficacy. For example, mutants lacking PncA, which converts PZA into its active pyrazinoic acid form (14), were highly overrepresented in the treated mice, highlighting the singular importance of PZA in the activity of this regimen (Fig. 1D). In addition, we found that mutations in *mmaA1*, *mmaA2*, and *cmaA2* sensitized the bacterium to drug treatment. All of these genes encode functions necessary for mycolate modification, and chemical inhibition of these partially redundant activities has been shown to increase cellular permeability to antibiotics (15). Additional protein families associated with cell wall structure altered drug efficacy. PE/PPE family members have been implicated in cell envelope integrity (16), and we found that mutations in the *ppe50-ppe51* pair increased killing (Fig. 1E). Conversely, the loss of enzymes (*ppsA*, *ppsC*, and *drrA*) necessary for the synthesis of the major cell envelope lipid, phthiocerol dimycocerosate, decreased clearance. Drug access appeared to be similarly limited by multiple classes of efflux pumps, as mutations in members of the ATP-binding cassette (*rv1747*), major facilitator superfamily (*rv3728*), and MmpL (*mmpL8* and *mmpL10*) families were found to increase bacterial clearance (Table S2).

In addition to these known mechanisms, we identified a number of novel functions that altered bacterial killing. Prominent among these were pathways involved in carbon metabolism. For example, mutation of both assayable steps of the glycogen synthetic pathway (*glgA* and *glgC*) increased antibiotic activity (Fig. 1F). This pathway promotes carbon storage through carbohydrate anabolism, a general process that has been previously implicated in drug tolerance (6). In addition, we identified the *glpK* gene, which encodes the *sn*-glycerol-3 kinase of M. tuberculosis. Twenty-six of the 29 insertional mutants in this gene showed a similar decrease in clearance rate upon drug treatment (Fig. 1G). When pools isolated before and after treatment were directly compared, only *pncA* mutations produced a statistically significant reduction that was greater than those in *glpK* (Fig. 1H). This relatively dramatic phenotype was explored in more detail.

### Glycerol metabolism increases drug efficacy *in vitro* and during murine infection.

**FIG 2:**
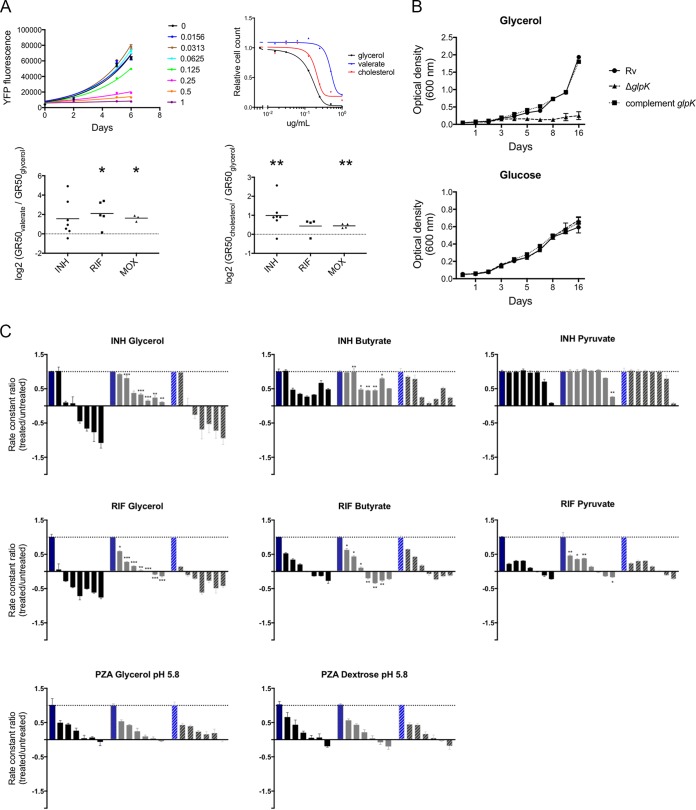
Glycerol metabolism broadly increases drug efficacy *in vitro*. (A, top left) Growth of H37Rv on cholesterol and treated with moxifloxacin at the indicated concentrations. Growth was measured by yellow fluorescent protein (YFP) fluorescence. (Top right) GR_50_ for moxifloxacin (MOX) in medium containing either glycerol, valerate, or cholesterol. (Bottom) GR_50_ ratios for INH (circles), RIF (squares), and MOX (triangles) grown on different carbon sources. Shown are valerate/glycerol (left) and cholesterol/glycerol (right). Significance was calculated using one-sample *t* test with a theoretical mean value of 0: *, *P* = 0.05; **, *P* = 0.01. (B) Growth kinetics of H37Rv (circles), △*glpK* (triangles), and complement (squares) strains on glycerol (top) or glucose (bottom). Plotted means from 3 biological replicates with standard deviations are shown. (C) Growth of H37Rv (black bars), Δ*glpK* (gray bars), and complement *glpK* (striped bars) strains after treatment with INH or RIF in media containing glycerol, butyrate, or pyruvate and PZA in media containing glycerol or dextrose at pH 5.8. Growth was assessed by the growth constant, *k*, normalized to no-antibiotic controls and plotted as ratios (treated/untreated), where 1 is the growth constant without antibiotic (indicated by a dotted line). Antibiotic concentrations started at 2 μg/ml, 1 μg/ml, and 400 μg/ml for INH, RIF, and PZA, respectively, and were serially diluted 2-fold for 6 dilutions. Significance was calculated using an unpaired *t* test with Benjamini-Hochberg multiple-testing correction. *, *P* = 0.03; **, *P* = 0.002; ***, *P* = 0.0002; ****, *P* < 0.0001.

GlpK is responsible for phosphorylating the 3-position of glycerol, which is necessary for its catabolism via the lower glycolytic pathway. To determine if glycerol catabolism *per se* was capable of enhancing the activity of TB drugs, the effects of INH, RIF, and moxifloxacin (MOX) were compared in media containing glycerol or other carbon sources known to be used during infection, fatty acid and cholesterol (17, 18). These carbon sources supported different growth rates, which can confound endpoint-based determinations of antibiotic activity, such as standard MIC measurements. As a result, we quantified the growth rate (GR) of bacteria over a time course and determined the concentration of each drug that was necessary to decrease this rate by 50%, which is expressed as GR_50_ (19). Using this approach, we found that glycerol catabolism produced a modest but reproducible decrease in the GR_50_ for several drugs. Growth in glycerol significantly increased the efficacy of RIF and MOX compared to growth in valerate and enhanced the efficacy of INH and MOX compared to growth in cholesterol (Fig. 2A).

To further investigate the role of glycerol metabolism in drug efficacy, a *glpK* deletion mutant of M. tuberculosis was constructed. The Δ*glpK* mutant was unable to grow in media containing glycerol as the sole carbon source (Fig. 2B), indicating that the deleted gene encodes the sole glycerol-3 kinase activity. The effect of antibiotics on the growth rates of *glpK*-sufficient and *glpK*-deficient strains was then compared in media containing different carbon sources. When glycerol was present in the medium, the Δ*glpK* mutant was significantly less sensitive to INH and RIF than the wild type or the complemented mutant (Fig. 2C). This difference largely disappeared when glycerol was replaced with either the nonglycolytic substrate, butyrate, or a glycolytic product that bypasses the triose phosphate pool, pyruvate. The differential effects of these carbon sources indicated that the assimilation of exogenous glycerol was primarily responsible for *glpK*’s influence on drug sensitivity. PZA sensitivity was assessed at pH 5.8 to maximize the *in vitro* efficacy of the drug. However, under these conditions, *glpK* deletion did not alter PZA sensitivity. As INH, RIF, and MOX have distinct mechanisms of action, the effect of glycerol catabolism on antibiotic activity *in vitro* did not appear to be specific to a particular drug or target pathway.

**FIG 3:**
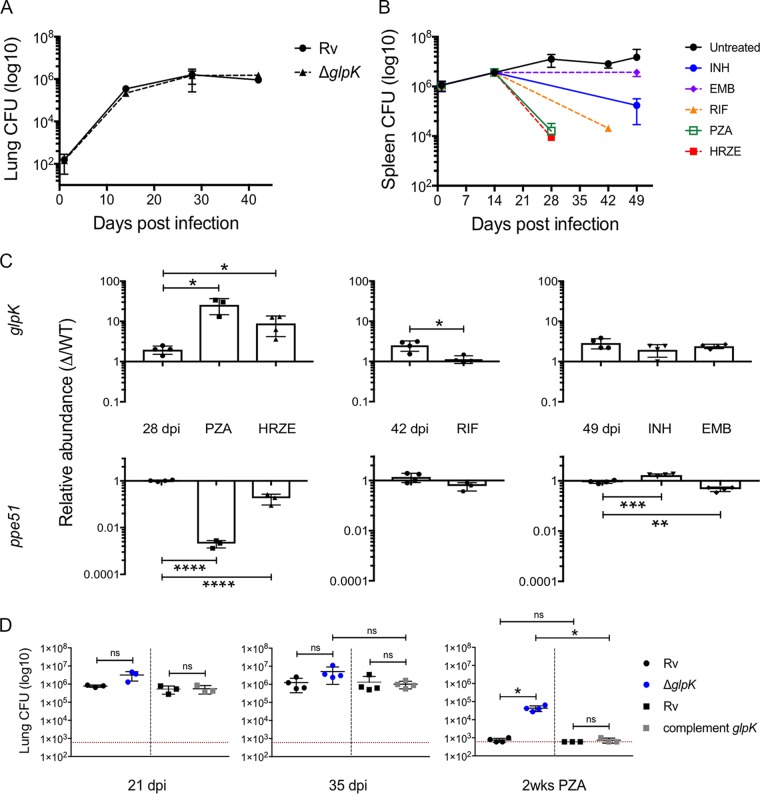
Loss of glycerol kinase increases survival under PZA treatment *in vivo*. (A) Lung CFU of H37Rv (circles) and △*glpK* (triangles) strains from BALB/c mice after aerosol infection with a dose of 500 to 700 CFU/mouse. Shown are plotted means from 4 biological replicates with standard deviations. (B) Spleen CFU from BALB/c mice after intravenous infection with pooled mutant strains both untreated (black circles) and treated with the indicated antibiotic. Plotted means from 4 biological replicates with standard deviations are shown. (C) Relative abundance of Δ*glpK* (top) and Δ*ppe51* (bottom) strains compared to that of the wild type *in vivo* after antibiotic treatment. Treatment times were 14 days for PZA and MIX, 28 days for RIF, and 35 days for INH and EMB. Individual points are biological replicates normalized to day 0 ratios. Significance was calculated using unpaired *t* test with Benjamini-Hochberg multiple testing correction: *, *P* = 0.03; **, *P* = 0.002; ***, *P* = 0.0002; ****, *P* < 0.0001. (D) Lung CFU of H37Rv, Δ*glpK*, and complement strains from BALB/c mice after aerosol infection and treatment with PZA. Data represent two competition infections: 1:1 H37Rv and Δ*glpK* (black and blue circles, respectively) strains, dose of 700 to 1,000 CFU/mouse, and 1:1 H37Rv and complement (black and gray squares, respectively) strains, dose of 300 to 500 CFU/mouse. Treatment with PZA was started at 21 dpi and continued to 35 dpi. Shown are plotted means and standard deviations, and individual points are biological replicates. Limits of quantification are indicated by dotted red lines. Significance was calculated using unpaired *t* test with Benjamini-Hochberg multiple testing correction: *, *P* = 0.03; **, *P* = 0.002; ***, *P* = 0.0002; ****, *P* < 0.0001; ns, not significant.

The mouse model was then used to explore the role of *glpK* during infection. Consistent with both our TNseq data and previous work (20), deletion of the *glpK* gene did not affect the growth or persistence of M. tuberculosis in the lungs of mice after aerosol infection (Fig. 3A). To quantify the effect of *glpK* deletion on the efficacy of individual drugs, mice were inoculated via the intravenous route with a mixture of wild-type and Δ*glpK* bacteria and treated with antibiotics, as was done for the initial TNseq screen. Another mutant lacking the *ppe51* gene, which TNseq predicted to be hypersensitive to multidrug treatment (Fig. 1E), was included as an additional control. Since this study did not require maintaining the complex mutant mixture needed in the TNseq study, more prolonged treatment regimens could be used. As we observed previously, all drug regimens reduced the bacterial burden, and PZA or combination therapy had the greatest effect (Fig. 3B). Surviving bacteria were recovered by plating at the indicated time points, and the relative abundance of the three M. tuberculosis strains was determined by quantitative PCR. Both mutants demonstrated the predicted phenotypes in animals treated with the four-drug combination therapy for 2 weeks, as the Δ*glpK* mutant was cleared significantly more slowly and the Δ*ppe51* mutant significantly more rapidly than the wild type (Fig. 3C). These phenotypes were even more pronounced in animals treated with PZA alone. In contrast to the broadly sensitizing effect of glycerol catabolism *in vitro*, the Δ*glpK* mutant behaved similarly to the wild type in mice treated with RIF, INH, or EMB. The decreased efficacy of PZA against the Δ*glpK* mutant was also found in the lungs of mice infected via aerosol. In this model, treatment with PZA between 21 and 35 days postinfection reduced the bacterial burden of wild-type and Δ*glpK* complemented strains by at least 1,000-fold but had a significantly reduced effect on the *glpK*-deficient mutant (Fig. 3D). Thus, while glycerol catabolism can nonspecifically alter antibiotic susceptibility *in vitro*, *glpK* deletion preferentially reduced the effect of PZA and a PZA-containing multidrug regimen in this animal model.

### Glycerol catabolic defects are associated with extensive drug resistance in Korea.

**FIG 4:**
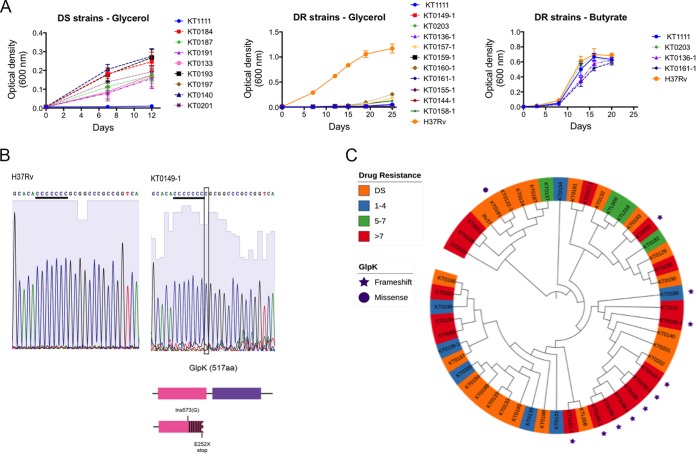
Glycerol catabolic mutations associated with XDR strains. (A) Growth kinetics of drug-susceptible (DS) and drug-resistant (DR) clinical isolates on glycerol- or butyrate-containing media. Shown are plotted means from 3 biological replicates with standard deviations. (B) Sanger sequencing of *glpK* from H37Rv and clinical isolate KT0149-1. The homopolymer region is in the first domain of the protein. One-bp insertion changes downstream amino acid sequence and introduces a premature stop codon at amino acid 252. (C) Phylogenetic tree of M. tuberculosis isolates from Korea with various drug susceptibility profiles: DS (orange); DR, 1 to 4 antibiotics (blue); DR, 5 to 7 antibiotics (green); and DR >7 antibiotics (red). Mutations in *glpK* gene are indicated: frameshift mutations, purple stars; missense mutations, purple circles.

As the *glpK* deletion did not alter bacterial fitness during infection and conferred a benefit upon drug treatment, we hypothesized that mutations altering glycerol catabolism are positively selected during the evolution of drug resistance in natural populations. As a first test of this hypothesis, we characterized a panel of Korean M. tuberculosis isolates that varied in drug sensitivity profiles, from fully sensitive strains to extensively evolved clones that were phenotypically resistant to more than ten different antibiotics (see Table S3 for strain characteristics). To investigate whether glycerol catabolic defects were selected during the evolution of resistance in these strains, we subcultured a random subset of drug-sensitive or extensively resistant isolates in media containing glycerol as the sole carbon source. Drug-sensitive strains grew at a rate similar to that of a standard laboratory strain (H37Rv). However, while the extensively drug-resistant isolates could grow in butyrate, none of the tested isolates could grow in the glycerol-containing media (Fig. 4A and Table S3).

10.1128/mBio.00663-19.3 TABLE S3 Phenotypic drug sensitivity profiles of Korean M. tuberculosis isolates. Strain characteristics of Korean M. tuberculosis isolates. ND, not determined. Beijing (K), Korean clade of the Beijing lineage. Download Table S3, XLSX file, 0.02 MB. Copyright © 2019 Bellerose et al. 2019 Bellerose et al. This content is distributed under the terms of the Creative Commons Attribution 4.0 International license.

The whole-genome sequences (WGS) of these isolates were determined. Based on WGS, this collection was predominantly comprised of a Korean sublineage of East Asian strains (21), and the multidrug-resistant (MDR) phenotypes could generally be attributed to known high-level resistance-conferring mutations (Table S4). Inspection of the WGS data revealed that the glycerol catabolic defect in 9 of the 11 tested strains could be attributed to loss-of-function mutations in the *glpK* gene. These strains all harbored a one-base expansion of the same homopolymeric sequence (GGGGGGG) in the 5′ half of the *glpK* open reading frame. The sequence of the *glpK* homopolymer was verified in the entire panel by targeted Sanger sequencing (Fig. 4B). This mutation is predicted to eliminate GlpK enzymatic activity, as it introduces a premature termination codon that eliminates the majority of the open reading frame, and the same homopolymer expansion has been previously observed in M. bovis strains lacking glycerol kinase activity (22). An additional missense mutation altering amino acid 169 was identified in an otherwise *glpK* wild-type allele, but the functional significance of this mutation is unclear. Two of the phenotypically glycerol-deficient strains carried no obvious mutations in *glpK* or other glycerol catabolic genes. Thus, while *glpK* frameshifts appear to be the most common lesion associated with glycerol catabolic defects in this collection, other mechanisms contribute in a fraction of isolates.

10.1128/mBio.00663-19.4 TABLE S4 SNPs in known resistance-associated genes from Korean M. tuberculosis isolates. SNPs within known resistance-conferring genes for individual Korean M. tuberculosis isolates. Known resistance-associated mutations were derived from A. Sandgren, M. Strong, P. Muthukrishnan, B. K. Weiner, et al., PLoS Med 6:e2, 2009, https://doi.org/10.1371/journal.pmed.1000002. Occurrences, number of times the indicated SNP is identified in the strain panel (colored by frequency of occurrence). Drug, antibiotic resistance associated with the indicated SNP. DR score, qualitative assessment of the extent of drug resistance based on drug class. DR phenotype, common clinical classification of DR phenotype. All, number of individual antibiotics to which each strain is resistant; 0, no polymorphism found relative to drug-sensitive strains; 1, polymorphism found. Download Table S4, XLSX file, 0.1 MB. Copyright © 2019 Bellerose et al. 2019 Bellerose et al. This content is distributed under the terms of the Creative Commons Attribution 4.0 International license.

All ten of the *glpK* frameshifts identified in this panel were found in multidrug-resistant strains, particularly the highly evolved strains that were resistant to more than eight different drugs (Fig. 4C). Based on the phylogenetic relationship between these strains, the identified *glpK* frameshifts represent at least three independent mutational events. Alternative tree topologies necessary to accommodate fewer mutational events were significantly less likely (*P* < 10^−4^). Thus, in this relatively small collection of strains, inactivating mutations in *glpK* were frequent and associated with defective glycerol utilization and drug resistance.

### GlpK frameshift mutations are common in M. tuberculosis isolates and associated with drug resistance in Peru.

**FIG 5:**
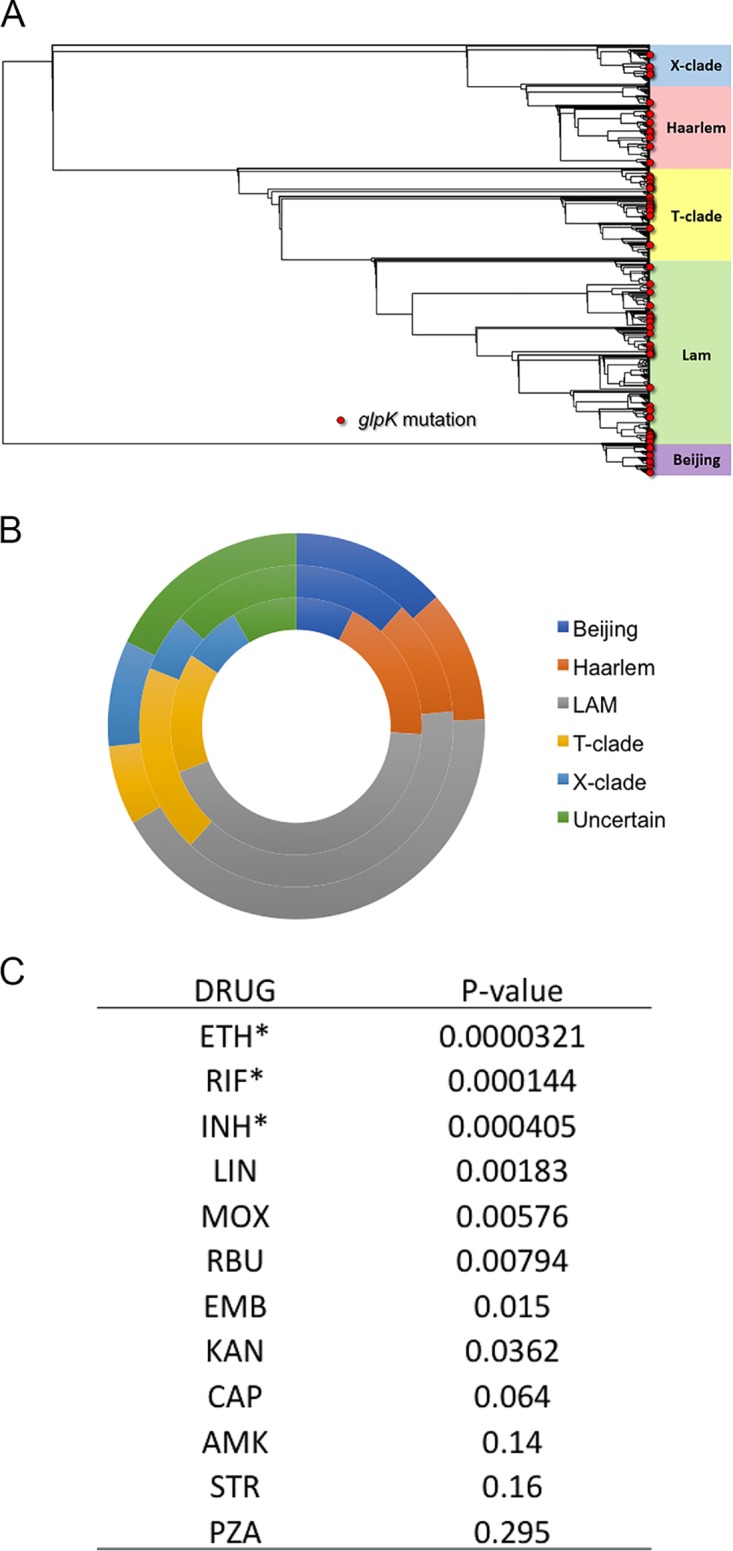
GlpK mutations associated with drug resistance in clinical isolates from Peru. (A) Phylogenetic tree of M. tuberculosis isolates from Peru. GlpK mutations are indicated (red circles). (B) Representation of GlpK mutations in different lineages: lineage distribution of 1,031 GWAS samples (outer); distribution of 68 *glpK* mutations (middle); distribution of 45 single-base expansions, T57GT, of the *glpK* homopolymer (inner). (C) Association between *glpK* mutations and drug resistance. Statistical significance (*) based on Bonferroni correction with a type 1 error rate of 0.01. INH, isoniazid; RIF, rifampin; RBU, rifabutin; EMB, ethambutol; PZA, pyrazinamide; STR, streptomycin; LIN, linezolid; MOX, moxifloxacin; AMK, amikacin; KAN, kanamycin; CAP, capreomycin; ETH, ethionamide.

Frameshift mutations in homopolymeric DNA sequences can represent high-frequency and reversible events (10). To assess the frequency of this mutation in a larger population and to further explore its association with drug resistance, we analyzed the whole-genome sequences of a larger collection of isolates from Peru. Of 1,031 sequenced strains, 68 isolates harbored nonsynonymous variants in the *glpK* gene. Of these, 45 contained a single-base expansion of the *glpK* homopolymer and 2 contained a two-base expansion. These frameshifts were found in all phylogenetic clades of M. tuberculosis, indicating that this mutation has arisen through multiple distinct mutational events in different lineages of the pathogen (Fig. 5A and B). In total, homopolymer expansion accounted for 66% of the nonsynonymous *glpK* variants, and 4.6% of all isolates harbored frameshift mutations disrupting the *glpK* open reading frame.

As we found in the smaller set of Korean strains, *glpK* frameshifts were significantly associated with drug resistance. No instances of *glpK* frameshifts were found in the 90 phenotypically drug-sensitive strains. In contrast, 44 of the 739 isolates that met the WHO criteria for MDR carried these mutations (*P* = 3 × 10^−5^). In this large collection, we were also able to test the association between *glpK* genotype and resistance to individual drugs and found significant associations with RIF, INH, and ethionamide (ETH) (Fig. 4C). The lack of observed association with PZA resistance could have been due to the unreliability of this phenotypic assay. Indeed, using a genotypic assay we found a significant association between *glpK* frameshifts and nonsynonymous *pncA* variants (*P* = 0.001). Thus, the *glpK* homopolymer is the site of the majority of variation in this gene, and expansion of this hypervariable region is associated with the evolution of drug resistance.

## DISCUSSION

Drug tolerance has been proposed to contribute to both relapsing TB disease and the emergence of drug-resistant clones (23). Most current models to explain tolerance in mycobacteria are largely restricted either to nonheritable processes, such as changes in gene expression or functionally asymmetric cell division, or stably heritable mutations (24). Our data provide a new mechanism by which alterations in a hypervariable region in the *glpK* gene produces bacteria that persist during antibiotic treatment and could contribute to the emergence of drug-resistant clones.

Insertions and deletions in a homopolymeric region of an open reading frame is a common mechanism to produce high-frequency reversible phenotypic variation in bacteria. These mutations are generally thought to result from slipped-strand mispairing during DNA replication. However, additional DNA repair mechanisms, such as mismatch repair (25) or base excision repair (26), can alter the frequency and directional bias of the process. The exclusive bias for +1 and +2 frameshifts in the *glpK* gene of clinical isolates argues for a more complex process than simple replicative error, and the ultimate frequency of mutants may also be influenced by the specific fitness effect of each frameshift. As a result, it is difficult to anticipate the rate of variation that occurs during infection. Regardless, we identified +1 and +2 frameshifts in 6% of the Peruvian MDR isolates. Since clinical samples are routinely cultured in glycerol-containing media, which would be expected to select for reversion to the wild-type *glpK* coding sequence, it is likely our data underestimate the frequency of *glpK* mutants. The observed prevalence of this mutation is clearly high enough to produce a significant population of *glpK*-deficient clones that alter drug efficacy.

Our studies in the mouse model demonstrate that *glpK*-deficient bacteria are drug tolerant during infection. The mechanism(s) that underlies the drug tolerance of *glpK*-deficient bacteria is likely to be complex. Common fates for glycerol-3-phosphate are catabolism via the lower glycolytic pathway, incorporation into anabolic pathways, and spontaneous degradation to methylglyoxal. As a result, glycerol assimilation can alter growth rate, metabolism, and cellular structure. While not yet conclusive, our *in vitro* studies argue against some of these mechanisms. Both the *in vitro* effects of glycerol supplementation and the *in vivo* effects of *glpK* expression were independent of growth rate (Fig. 1 and 2), and the differential effect of *glpK* in glycerol versus pyruvate growth media indicates that glycolytic flux *per se* is not the major determinant of drug efficacy. Thus, we speculate that the abundance of the triose phosphate pool or some derivative of this pool is primarily responsible for the general enhancement of antibiotic efficacy that we observed upon glycerol assimilation *in vitro*.

The nonspecific effect of *glpK* on multiple drugs that we observed *in vitro* is consistent with previous studies that identified frameshifts in the *glpK* homopolymer in mutants selected for spontaneous resistance to investigational antimycobacterial compounds *in vitro* (20). Similarly, *glpK* mutations have also been found in conjunction with additional mutations in strains selected to be drug tolerant (27) or PZA resistant (28) *in vitro*. Despite these relatively general effects on drug activity *in vitro*, *glpK* deletion preferentially reduced the efficacy of PZA-containing regimens in the mouse model used in this study. This apparent discrepancy could reflect differences in drug exposure, bacterial physiology, or GlpK functions in these two settings. The poor activity of PZA *in vitro*, where we observed no effect of *glpK* on PZA activity, makes it difficult to dissect these mechanisms in a more controlled system. Regardless, the identification of mutations that affect PZA efficacy only during infection highlights the importance of performing the original TNseq screen in an animal model.

The prevalence of *glpK*-deficient strains in natural populations and the preferential survival of these bacteria in drug-treated animals suggested that *glpK*-deficient clones contribute to the persistence of M. tuberculosis during therapy and provide precursors for the emergence of clones with high-level resistance-conferring mutations. It is unlikely that the effects of *glpK* variation would be noted in standard phenotypic drug susceptibility testing (DST). This situation is similar to common variation in the *prpR* gene, which specifically influences drug tolerance but not DST results (24). These observations raise the possibility that genotypic tests for common drug tolerance-inducing variants could predict treatment failure and eventually be used to tailor therapy. We note that our TNseq study identified a number of additional loss-of-function mutations that alter drug efficacy, and the genome contains more than 100 genes with homopolymeric regions that are at least as long as the one found in *glpK* (29). Together, these observations suggest that many phenotypically distinct subpopulations arise via similar mechanisms and influence antibiotic efficacy.

## MATERIALS AND METHODS

### Transposon sequencing.

BALB/cJ (stock no. 000651) mice were purchased from the Jackson Laboratory (Bar Harbor, ME, USA). Housing and experimentation were in accordance with the guidelines set forth by the Department of Animal Medicine of University of Massachusetts Medical School and Institutional Animal Care and Use Committee and adhered to the laws of the United States and regulations of the Department of Agriculture. Eight- to 12-week-old female animals were infected with 10^6^ CFU of a *himar1* transposon library (30) via the intravenous route. Groups of mice were treated with antibiotics starting at 14 days postinfection. Antibiotics were administered via drinking water at the following concentrations: 0.1 g/liter isoniazid (Sigma), 0.6 g/liter ethambutol (Sigma), 0.1 g/liter rifampin (Sigma), and 15 g/liter pyrazinamide (Sigma). At the indicated time points, mice were sacrificed, spleens and lungs were isolated and homogenized, and CFU numbers were determined by plating dilutions on 7H10 agar with 10 μg/ml kanamycin. For library recovery, approximately one million CFU per mouse were plated on 7H10 agar with kanamycin (10 μg/ml). Genomic DNA was extracted (13), and the relative abundance of each mutant was estimated as described previously (13). Statistical analysis of log_2_ fold change (log_2_FC) in counts between conditions (two-way analysis) was performed by resampling (31). The three-way analysis measures the difference in log_2_FC (Δlog_2_FC) measured under two selective conditions relative to a common starting condition: Δlog_2_FC = log_2_FC(condition 1) − log_2_FC(condition 2).

In the present case, condition 1 was 14 days postinfection plus 7 days of antibiotic treatment, condition 2 was 21 days postinfection, and the starting condition was 14 days postinfection (the start of drug treatment). Statistical significance was assessed by resampling. For each gene, the sampling distribution of Δlog_2_FC was obtained by resampling with replacement of the insertion counts at each TA within the gene (after normalization across all libraries). Counts for replicates were pooled prior to resampling. For each of 10,000 resamples, Δlog_2_FC was calculated. The *P* value was taken as the fraction of the cumulative frequency distribution of Δlog_2_FC falling outside Δlog_2_FC = 0, on the negative side for values measured as Δlog_2_FC > 0, or on the positive side for values measured as Δlog_2_FC < 0 (equivalent to a 1-tailed test). The resulting *P* values were adjusted for multiple testing by Benjamini-Hochberg false discovery rate.

### M. tuberculosis gene deletion and Δ*glpK* mutant characterization.

M. tuberculosis H37Rv was maintained in Middlebrook 7H9 medium containing oleic acid-albumin-dextrose-catalase (OADC), 0.2% glycerol, and 0.05% Tween 80 and grown with shaking (200 rpm) at 37°C. Hygromycin (50 μg/ml) or kanamycin (20 μg/ml) was added when necessary. All work with M. tuberculosis adhered to the CDC-NIH *Guide for Biosafety in Microbiological and Biomedical Laboratories* (32). *glpK* and *ppe51* were deleted by allelic exchange as described previously (33), and this work adhered to NIH guidelines for research involving recombinant DNA molecules. Nucleotides 4138237 to 4139720 (*glpK*) or 3501829 to 3502901 (*ppe51*) were replaced by the vector pKM464, including qTag-22 or qTag-26 (34) for *glpK* and *ppe51*, respectively. The Δ*glpK* strain was cultured in glycerol-free 7H9. Glycerol-dependent growth was assessed in minimal medium containing asparagine (0.5 g/liter), KH_2_PO_4_ (1 g/liter), Na_2_HPO_4_ (2.5 g/liter), ferric ammonium citrate (50 mg/liter), MgSO_4_·7 H_2_O (0.5 g/liter), CaCl_2_ (0.5mg/liter), ZnSO_4_ (0.1mg/liter), 0.1% tyloxapol, and either 0.1% glycerol or 0.1% dextrose. For *in vitro* antibiotic susceptibility testing, isoniazid and rifampin were used at 2 and 1 μg/ml, respectively, and serially diluted 2-fold. Bacteria were inoculated to a starting optical density at 600 nm (OD_600_) of 0.05 in 96-well plates with 7H9 medium containing OADC, 0.05% Tween 80, and 0.2% glycerol, butyrate, or pyruvate. Pyrazinamide was used at 400 μg/ml and serially diluted 2-fold. Bacteria were inoculated to a starting OD_600_ of 0.01 in inkwells containing 7H9 medium supplemented with OADC, 0.2% glycerol, and 0.05% tyloxapol at pH 5.8 and grown with shaking. Growth was monitored by OD_600_. Conditions were assessed in triplicate. Antibiotic efficacy was determined by comparing growth rate under increasing drug concentrations. OD_600_ was plotted and the rate constant (*k*) value was determined for all conditions using an exponential growth model. Rate constants posttreatment were normalized to levels for no-antibiotic controls.

Δ*glpK* mutant fitness *in vivo* was determined by inoculating mice with a 1:1 mixture of Δ*glpK* (hygromycin resistant) and H37Rv (harboring pJEB402 chromosomally integrated plasmid encoding kanamycin resistance) strains via the aerosol route. At the indicated time points, mice were sacrificed and CFU numbers in spleen and lung homogenate were determined by plating on 7H10 agar. Fitness in the presence of antibiotic was assessed by a similar competitive assay. Mice were infected with a pool of strains at equal ratios via the intravenous route (10^6^ total CFU/mouse). Groups of mice were treated with antibiotics starting at 14 days postinfection, as described for the TNseq study. At the indicated time points, approximately 10,000 CFU from the spleen homogenate of each mouse were plated on 7H10 agar. Genomic DNA was extracted for quantitative real-time PCR analysis (34). Briefly, the abundance of the constant and variable regions of the q-Tag present in each mutant was determined by TaqMan PCR assay, as described previously (34), and used to calculate a variable/constant region ratio for each strain. The abundance of each mutant strain was then plotted relative to that of wild-type H37Rv (mutant/wild type). Values were normalized to initial day 0 ratios.

### GR_50_ determination.

Bacteria were grown in minimal medium with 0.1% glycerol, 0.1% valeric acid, or 0.1% cholesterol on 96-well plates. Isoniazid, rifampin, and moxifloxacin were used at 1, 0.062, and 1 μg/ml, respectively, and serially diluted 2-fold. A no-antibiotic control was included in each experiment. Bacteria were inoculated to a starting OD_600_ of 0.05, and growth was monitored by OD_600_ and fluorescence. Conditions were prepared in triplicates. Antibiotic efficacy was determined by growth rate inhibition. The exponential growth constant (*k*) value was determined for all conditions. The *k* value of each antibiotic concentration was normalized to the *k* value of the no-drug control. The GR_50_ value was determined as the concentration of antibiotic that resulted in a 50% decrease in growth rate, as previously described (19).

### Phenotypic and genotypic analysis of Korean strains.

Strains were collected from the National Culture Collection for Pathogens, which is maintained by the Korea Centers for Disease Control and Prevention. Phenotypic DST testing for each strain was conducted by an absolute concentration method using Löwenstein-Jensen agar with critical concentrations of TB drugs (in μg/ml): isoniazid (0.2), rifampin (40), ethambutol (2), streptomycin (10), kanamycin (40), prothionamide (40), cycloserine (30), para-aminosalicylic acid (1), ofloxacin (2), pyrazinamide (50; pH 4.65), capreomycin (40), moxifloxacin (2), amikacin (40), levofloxacin (2), *p*-nitrobenzoic acid (500), and rifabutin (40). Strains were classified as resistant if drug-containing media produced more than 1% of the CFU observed in control cultures. To test growth on glycerol, M. tuberculosis was grown in Middlebrook 7H9 broth with 0.5% glycerol, 0.05% tyloxapol, catalase, and fatty acid-free bovine serum albumin (Sigma). Inocula were cultivated in 7H9-OADC-Tween 80 to an OD of 0.1 to 0.2, washed with phosphate-buffered saline plus tyloxapol (0.05%), and diluted to ∼10^6^ CFU/ml.

Genomic DNA was sequenced either by Ion Torrent (yielding an average read length of 170 bases) or Illumina (300-base paired-end reads) platforms. In both cases, reads were aligned using bwa mem (version 0.7.12) against Mycobacterium tuberculosis H37Rv reference GenBank accession no. NC_018143.2. Variants were called using GATK 3.3-0 (35, 36) by following the developer’s best practices: (i) picard 1.96 MarkDuplicates, (ii) GATK Realigner Target Creator, (iii) GATK IndelRealigner, (iv) GATK BaseRecalibrator, (v) GATK UnifiedGenotyper, and (vi) GATK GenotypeGVCFs. Base recalibration was performed iteratively using the initial Ion variant calls, obtained without recalibration, to obtain a set of polymorphic sites for use in step 4. Final filtering was performed separately for single-nucleotide polymorphism (SNP) and insertion/deletion (indel) calls: for SNPs, FS of >60.0, MQ of <40.0, MQRankSum of <−12.5, and ReadPosRankSum of <−8.0; for indels, FS of >200.0 and ReadPosRankSum of <−20.0. Call-passing filters were combined and a final filter QD of <20.0 was applied. Only calls passing all filters were combined into the final vcf file (a total of 7,418 variants). For phylogenetic analysis, only variant SNPs were used. The final alignment (which included the corresponding NC_181843.2 reference bases) consisted of 51 taxa × 7,247 positions. A maximum likelihood phylogeny was obtained using PHYML version 20120412 (37) with the generalized time-reversible model. Trees were visualized with iTOL (38). Alternative tree topologies were generated using PHYLIP retree (39), and SH tests were performed using PAML baseml, version 4.8 (40).

### Phenotypic and genotypic analysis of Peruvian strains.

Drug-resistant phenotypes were determined by measuring MICs to 12 antituberculosis drugs. For strains found to be sensitive at the critical concentration recommended by the WHO for each drug (41), we tested two MIC levels below the critical concentration, and for those resistant at the critical concentration, we tested six levels above it. The testing concentrations deviated from the traditional doubling in order to better detect intermediate-level MICs that are theoretically achievable levels in patient sera.

Strains were sequenced on the Illumina platform to produce 100 to 150 paired-end reads and coverage of at least 50-fold. The paired-end raw sequence data were mapped to the H37Rv reference genome using the BWA mem algorithm. We used SAMtools (default settings) (42) and pilon (43) to identify single-nucleotide variants and insertions and deletions up to approximately 100 bp using a coverage-based approach. We assigned a variant call as missing if the valid depth of coverage at a specific site was less than 10 reads, if the mean read-mapping quality at the site did not reach 7, or if none of the alternative alleles accounted for at least 90% of the valid coverage. The genotype of *glpK* was determined by a binary burden score that represented the presence of any nonsynonymous single-nucleotide variant, insertion, or deletion observed in that gene. M. tuberculosis genetic lineages were determined using a previously published SNP barcode (44). A neighbor-joining tree was derived using https://cran.r-project.org/web/packages/ape/index.html.

A linear mixed model was used to examine the associations between *glpK* genotype and the rank-transformed MICs phenotypes of 12 drugs and *pncA* genotype. We adjusted for the population structure using a genetic relatedness matrix (GRM), calculated from a pairwise distance matrix using synonymous single-nucleotide variants of the complete genome using the software GEMMA. The type I error rate was set at 0.01 after a Bonferroni correction accounting for the multiple comparisons. The linear mixed model was also performed using GEMMA (45).
